# Theoretical Prediction of the Sublimation Behavior by Combining Ab Initio Calculations with Statistical Mechanics

**DOI:** 10.3390/ma16072826

**Published:** 2023-04-01

**Authors:** Yang Hu, Kai Wang, Michael Müller, Egbert Wessel, Robert Spatschek

**Affiliations:** 1 Institute of Energy and Climate Research IEK-2, Forschungszentrum Jülich GmbH, 52428 Jülich, Germany; 2 JARA Energy, 52428 Jülich, Germany

**Keywords:** sublimation, statistical mechanics, ab initio modeling, thermodynamics

## Abstract

We develop a theoretical model to predict the sublimation vapor pressure of pure substances. Moreover, we present a simple monoatomic molecule approximation, which reduces the complexity of the vapor pressure expression for polyatomic gaseous molecules at a convincing level of accuracy, with deviations of the Arrhenius prefactor for NaCl and NaF being 5.02% and 7.08%, respectively. The physical model is based on ab initio calculations, statistical mechanics, and thermodynamics. We illustrate the approach for Ni, Cr, Cu (metallic bond), NaCl, NaF, ${\mathrm{ZrO}}_{2}$ (ionic bond) and ${\mathrm{SiO}}_{2}$ (covalent bond). The results are compared against thermodynamic databases, which show high accuracy of our theoretical predictions, and the deviations of the predicted sublimation enthalpy are typically below 10%, for Cu even only 0.1%. Furthermore, the partial pressures caused by gas phase reactions are also explored, showing good agreement with experimental results.

## 1. Introduction

The determination of the thermodynamic parameters of the solid-to-vapor (sublimation) phase transition is significant for chemical thermodynamics. Even if vapor pressures are often low, they can lead to mass loss and accompanying concentration change and resulting in modification of functional behavior. Additionally, the vapor can condense elsewhere and also affect the material behavior there. Consequently, sublimation processes are considered to be important for various applications in different scientific areas [1]. For example, the analysis of the sublimation behavior of cathode materials such as ${\mathrm{La}}_{0.58}{\mathrm{Sr}}_{0.4}{\mathrm{Co}}_{0.2}{\mathrm{Fe}}_{0.8}O_{3-\delta }$ (LSCF) in solid oxide fuel cells (SOFCs) and electrolytes can contribute to the reduction of their degradation, which is of high interest for green energy applications [2]. Furthermore, Liu et al. have determined the thermodynamic activities of SrO, FeO and CoO in $\mathrm{Sr}{\mathrm{Ti}}_{1-x-y}{\mathrm{Co}}_{x}{\mathrm{Fe}}_{y}O_{3-\delta }$ compounds by measurements of the fugacity, whereby SOFC degradation-related chemical reactions can be predicted. In general, the investigation of the sublimation process and expanding the thermodynamic database have huge potential to be applied in various fields, such as material stability, chemical thermodynamics, energy, and chemical engineering [3].

Among the experimental methods to determine vapor pressures is the Knudsen effusion mass spectrometer (KEMS), which offers the highest accuracy for vaporization studies under near equilibrium conditions [4]. Nevertheless, this method is mainly suitable for high-temperature applications to ensure sufficiently high partial pressures, requiring in particular extended calibration processes, and is not suitable for all materials, hence the information in existing thermodynamic databases is still insufficient [5]. Therefore, to complement experimental methods, a theoretical and modeling perspective is desirable, which also helps to understand the details of the sublimation process.

For many years, electronic structure density functional theory (DFT) has played a great role in computational materials science, by solving the Kohn–Sham equations to obtain self-consistent electronic densities and to find ground-state energy. Initially, DFT calculations were designed for the prediction of ground-state energy (at 0 K), allowing the prediction of electronic, optical, mechanic, and thermodynamic properties of materials such as the density of states (DOS), band structure, second-harmonic generation coefficient, Young’s modulus, adsorption energy, chemical reaction activation energy, solvation energy, work function, etc.; see, e.g., [6,7,8]. Based on the DFT calculations, certain thermodynamic properties, phase transitions, and the influences of structural defects can be investigated [9]. Cervinka and Fulem investigated the sublimation enthalpy using DFT calculation [10]. Lopes Jesus et al. studied the sublimation enthalpy of the solid state of organic compounds also with the help of DFT calculations [11]. Furthermore, Halpern and Marzzacco used classical and statistical thermodynamics to calculate thermodynamic properties, such as the enthalpy and entropy of fusion and vaporization for water [12]. Zaby et al. used a modified partition function in quantum cluster equilibrium theory to predict the enthalpy and entropy of vaporization by employing statistical mechanics [13]. Thus, the analysis of the sublimation behavior by the combination of DFT calculation and statistical mechanics is promising and used for thermodynamic databases [14]. However, a pragmatic theoretical model which can predict the entire sublimation function for a solid is still lacking.

In this context, a reliable method combining ab initio calculations, statistical mechanics, and thermodynamics is desired and pursued in this article, which is solely dependent on theoretical calculations without any experimental or adjustable parameters. As such expressions can easily become involved, especially for complex molecules, simplifying expressions are desired, which allow for an easy, but still accurate prediction of vapor pressures. Therefore a pragmatic “monoatomic molecule approximation” is developed to simplify the polyatomic gas molecule calculations. This approximation can improve the calculation efficiency while maintaining the accuracy of the predictions and allows the reduction of the parameter requirements for the model. Although such a reduction may not be applicable for all cases, we demonstrate for a variety of molecules that the vapor pressure predictions are indeed reliable.

In our calculations, the gaseous phases are treated as ideal gases, and pure substances with different bonds are selected for demonstration purposes, i.e., Ni, Cr, Cu with a metallic bond, NaCl, NaF, ${\mathrm{ZrO}}_{2}$ with an ionic bond and ${\mathrm{SiO}}_{2}$ with a covalent bond. First, in our physical model, the cohesive energy $E_{\mathrm{coh}}$ of the solid phase is required as the difference between the solid and gaseous molecules, and it is obtained from DFT calculation using the Vienna Ab Initio Simulation Package (VASP) [15,16]. Second, we consider the different degrees of freedom of the molecules in the solid lattice and in the gas phase and construct their equilibrium conditions in the framework of statistical mechanics.

Atoms in crystalline solid phases vibrate with different frequencies in the lattice, and the vibration behavior is described by the phonon spectra [17]. These spectra are obtained via VASP with help of the Phonopy package, which is an open-source package for phonon calculations at harmonic and quasi-harmonic levels [18]. The K-path for the calculation of phonon spectra is obtained from the open-source package Vaspkit [19]. Instead of using the full spectra, we reduce the acoustic and optical branches to mean vibrational frequencies as central simplification.

Therefore, the article is organized as follows. In the next section, we develop expressions for the equilibrium vapor pressure and consider the possibility of further chemical reactions in the gas phase. Therefore, we first demonstrate the basic calculations given the complete molecule’s motions and then present the aforementioned monoatomic molecule approximation by considering only the translational degrees of freedom of the gaseous molecules. Consequently, the diatomic or triatomic gaseous molecules are treated similarly to the monoatomic gaseous molecules. Although this suppression is not appropriate, e.g., for the calculation of heat capacities, it turns out that it is sufficient for vapor pressure predictions. Afterward, we use statistical mechanics to connect the microstates to the thermodynamic properties. During sublimation, the exchange of particles between the solid phase and gaseous phases is possible, hence we use the equality of chemical potentials between the gas phase and the solid as an equilibrium condition. Moreover, further dissociation or following gas phase reactions are investigated. Here, we use ${\mathrm{Ni}}_{2}\left(g\right)$, ${\mathrm{Cu}}_{2}\left(g\right)$, Na(g), Cl(g), Zr(g) and $O_{2}\left(g\right)$ as examples to elucidate such situations. After these theoretical foundations, we present the computational details in the following section. The predictions are validated by comparing them to the commercial databases FactPS from the software package FactSage 8.1 [20]. Finally, we summarize and discuss the results of the physical model and elucidate the advantages and disadvantages in detail.

## 2. Theory

The sublimation behavior indicates the phase transition of the substance from the solid state to the gaseous state. During sublimation, the atoms vibrate in the lattice, and when the energy is large enough, the atoms are released from the lattice to become gaseous molecules. Typically, the vapor pressure *p* has an Arrhenius form, in the following denoted as sublimation function,

(1) $$\ln p=-\frac{\Delta H_{\mathrm{sub}}}{k_{B}T}+\ln A,$$

with *A* being a constant (prefactor). This relationship assumes that sublimation is a reaction with a single relevant activation barrier. The function shows a linear relationship between lnp and 1/T. Often, the sublimation enthalpy is given per mole of the substance, and then the slope of this line is defined as $\Delta H_{\mathrm{sub}}/R$ with the gas constant *R*. As we use here an atomic scale description, we use the equivalent normalization of energies per atom or molecule. Moreover, some molecules become unstable at a certain temperature, which leads to dissociation reactions. In our physical model, further possible chemical reactions are also considered. Thus, the main task for predicting the vapor pressure is the determination of the sublimation enthalpy $H_{\mathrm{sub}}$ and the prefactor *A*.

### 2.1. Sublimation Function

The calculation of the sublimation function is performed using statistical mechanics and DFT calculations. The canonical partition function Z for a system is given by [14,21,22]

(2) $$Z=\sum_{i}\exp\left(-\frac{E_{i}}{k_{B}T}\right),$$

where $E_{i}$ is the energy of the *i*th quantum state in the system and $k_{B}$ the Boltzmann constant, with the summation running over the entire phase space. The Helmholtz energy can be written as

(3) $$F=-k_{B}T\ln Z.$$

The chemical potential is obtained as

(4) $$\mu ={\left(\frac{\partial F}{\partial N}\right)}_{T,V}=-k_{B}T{\left(\frac{\partial \ln Z}{\partial N}\right)}_{T,V},$$

where *N* is the number of particles in this system. In case the Hamiltonian decomposes additively into independent contributions, the canonical partition function becomes

(5) $$Z=\prod_{i}z_{i}.$$

For a crystalline phase, $z_{i}$ can in particular be a vibrational oscillator. In general, the vibrational frequency of each oscillator is different and can be obtained from the phonon spectrum. The crystal structure has a significant influence on the phonon spectrum, consisting of acoustic and optical branches [17]. In our model, we use a simple harmonic approximation and anharmonic effects are ignored. Therefore, the expression for the partition function of single vibrational mode is [21]

(6) $$z_{s}=\frac{1}{2\sinh(\beta \hbar \omega /2)}.$$

Here, β is equal to $1/(k_{B}T)$, ℏ=h/2π is the reduced Planck constant, ω is the vibrational frequency of the considered mode, for which we will use in the following averaged values. More precisely, we take the average of the highest and lowest frequencies of both the acoustic and optic branches of the phonon spectrum.

Similarly, the partition function of gaseous phases is given by:

(7) $$Z_{g}=\frac{z_{g}^{N}}{N!},$$

where the factor 1/N! accounts for the usual indistinguishability of the atoms. In general, the gaseous molecule has different degrees of freedom, such as translational ($z_{\mathrm{trans}}$), rotational ($z_{\mathrm{rot}}$), and vibrational ($z_{\mathrm{vib}}$) degrees of freedom, and their expressions are [23]

(8) $$z_{\mathrm{trans}}={\left(\frac{2\pi mk_{B}T}{h^{2}}\right)}^{3/2}V,$$

(9) $$z_{\mathrm{rot}}=\frac{k_{B}T}{\sigma hc\tilde{B}},\;\left(\tilde{B}=\frac{\hbar }{4\pi cI},I=\mu r^{2}\right),$$

(10) $$z_{\mathrm{vib}}={\left(1-e^{-\beta hc\tilde{v}}\right)}^{-1}.$$

Here, *m* is the mass of the gaseous molecules, *V* is the volume, σ is the symmetry number of gaseous molecules, *c* is the light speed, $\tilde{B}$ is the rotational constant, *I* is the moment of inertia, μ is the reduced mass and *r* is the interatomic gas distance, and $\tilde{v}$ is the vibrational frequency of the gaseous molecules. The energy for the separation of electrons from the ground state is usually very large, thus, for most situations $z_{\mathrm{el}}=1$ is a suitable approximation. A significant exception is molecules with electronically degenerate ground states, for instance, the alkali metal atoms with $z_{el}=2$ [23].

The construction of the equilibrium state is then based on the chemical potential balance,

(11) $$\mu_{A(s)}=\mu_{A(g)}.$$

In this study, we exploit a physical model for the monoatomic molecule and polyatomic molecule first and then propose a simple approximation (monoatomic molecule approximation) that simplifies the expressions for practical applications.

#### 2.1.1. Monoatomic Molecule

A monoatomic gaseous molecule has only three translational degrees of freedom. For the solid phase, we use only the mean vibrational frequency of acoustic branches. Thus, the chemical potential of the molecule in the solid phase and gaseous molecules can be expressed as

(12) $$\mu_{s}=-k_{B}T\ln\left(z_{x}z_{y}z_{z}\right)+E_{\mathrm{solid}}=-3k_{B}T\ln z_{s}+E_{\mathrm{solid}}$$

with

(13) $$\mu_{g}=-k_{B}T\left(\ln\frac{z_{\mathrm{trans}}}{N}\right)+E_{gas}$$

From the chemical potential balance we obtain the expression for the vapor pressure in ideal gas approximation,

(14) $$p=\frac{{\left(k_{B}T\right)}^{5/2}{\left(2\pi m\right)}^{3/2}\exp\left(-\beta E_{\mathrm{coh}}\right)}{h^{3}\exp\left(-3\ln\left(\sinh\left(\beta \hbar \omega /2\right)\right)\right)},$$

which involves the cohesive energy $E_{\mathrm{coh}}=E_{\mathrm{gas}}-E_{\mathrm{solid}}$ per particle, which is obtained from ab initio simulations [24].

#### 2.1.2. Diatomic Molecule

For a diatomic molecule, we need to consider (i) the potential appearance of optical branches in the phonon spectrum of the solid phase, which are related to additional vibrational degrees of freedom, and (ii) the role of rotational and vibrational contributions in the gas phase. As a result, the vapor pressure can be written as

(15) $$p=\frac{{\left(k_{B}T\right)}^{5/2}{\left(2\pi m\right)}^{3/2}z_{\mathrm{rot}}\;z_{\mathrm{vib}}z_{\mathrm{el}}\exp\left(-\beta E_{\mathrm{coh}}\right)}{h^{3}\exp(-\sum_{i=1}^{6}\ln\left(\sinh\left(\beta \hbar \omega_{i}/2\right)\right)}.$$

In this paper, we use the mean frequency of acoustic and optic branches separately, thus, the resulting expression becomes

(16) $$p=\frac{{\left(k_{B}T\right)}^{5/2}{\left(2\pi m\right)}^{3/2}z_{\mathrm{rot}}z_{\mathrm{vib}}z_{\mathrm{el}}\exp\left(-\beta E_{\mathrm{coh}}\right)}{h^{3}\exp(-3\ln(\sinh(\beta \hbar \omega_{\mathrm{acoustic}}/2-3\ln\left(\sinh\left(\beta \hbar \omega_{\mathrm{optic}}/2\right)\right)},$$

where $\omega_{\mathrm{acoustic}}$ and $\omega_{\mathrm{optic}}$ are the mean frequencies of the acoustic and optical branches by taking the maximum and minimum value, respectively.

#### 2.1.3. Monoatomic Molecule Approximation

As the complexity of the description rises for larger molecules, a central question of the current paper is whether a simplified “monoatomic molecule approximation”, where the internal vibrational and rotational degrees of freedom of a molecule are ignored and it is treated as a point-like particle, is sufficient to deliver reliable results for the prediction of the vapor pressure. Essentially, this monoatomic molecule approximation ignores partially heat capacity contributions in both phases, i.e., the internal relative motion in the unit cell and the vibrational, rotational motion from the gaseous molecules. Therefore, a gaseous molecule is simplified to have only the transitional degrees of freedom. Additionally, for the crystalline phase, which contains different atom types inside its unit cell, only the acoustic branch of the phonon spectrum is retained.

This common simplification on both sides of the chemical potential balance equation offsets part of the deviations. Essentially, this simplification has an impact on the contribution of the heat capacity; however, the main part of the sublimation enthalpy is cohesive energy [10], and therefore we expect this approximation to deliver reasonable results.

### 2.2. Chemical Reaction

We use an $A_{m}B_{n}$ crystal as an example to clarify the complex sublimation behavior and its further chemical reaction. Aspects such as defect formation in the crystal are not considered, and we refer to [25] for further details. The gaseous molecule $A_{m}B_{n}\left(g\right)$ evaporates from the $A_{m}B_{n}\left(s\right)$ solid crystal, and then it dissociates into A(g) gas and B(g) gas atoms. According to Hess’s Law, the complete process is written as

(17) $$A_{n}B_{m}\left(s\right)\to \mathrm{nA}\left(g\right)+\mathrm{mB}\left(g\right).$$

In equilibrium, the change in Gibbs free energy ΔG must be equal to 0. According to Van’t Hoff’s equation ($\Delta G^{⊖}=-RT\ln K$), *K* is the equilibrium constant of the reaction. We therefore obtain

(18) $$\Delta G=nG_{A(g)}+mG_{B(g)}-G_{A_{n}B_{m}\left(g\right)}=\Delta G^{⊖}+RT\ln\left(\frac{\frac{p_{A(g)}^{n}p_{B(g)}^{m}}{{\left(p_{A(g)}^{⊖}\right)}^{n}{\left(p_{B(g)}^{⊖}\right)}^{m}}}{\frac{p_{AnBm(s)}}{p_{AnBm(s)}^{⊖}}}\right)=0.$$

Hence we obtain

(19) $$p_{A(g)}^{n}p_{B(g)}^{m}={\left(p_{A(g)}^{⊖}\right)}^{n}{\left(p_{B(g)}^{⊖}\right)}^{m}\frac{p_{\mathrm{AnBm}(s)}}{p_{\mathrm{AnBm}(s)}^{⊖}}\exp\left(-\frac{\Delta G^{⊖}}{RT}\right).$$

According to the balance of the chemical reaction we have $mp_{A(g)}=np_{B(g)}$, hence Equation (19) can be reformulated as

(20) $$p_{B(g)}={\left(\frac{m^{n}}{n^{n}}\right)}^{\frac{1}{m+n}}{\left(p_{A(g)}^{⊖}\right)}^{\frac{n}{m+n}}{\left(p_{B(g)}^{⊖}\right)}^{\frac{m}{m+n}}{\left(\frac{p_{\mathrm{AnBm}(s)}}{p_{\mathrm{AnBm}(s)}^{⊖}}\right)}^{\frac{1}{m+n}}\exp\left(-\frac{\Delta G^{⊖}}{(m+n)RT}\right)$$

with

(21) $$\Delta G^{⊖}=\Delta H^{⊖}+\Delta \left(TS^{⊖}\right).$$

Practically, we calculate the sublimation enthalpy at 0 K, and therefore $\Delta G^{⊖}=\Delta H^{⊖}$, hence

(22) $$\Delta H^{⊖}=nU_{A(g)}+mU_{B(g)}-U_{\mathrm{AnBm}(s)}=nE_{A(g)}+mE_{B(g)}-E_{\mathrm{AnBm}(s)}.$$

Then Equation (20) can be rewritten as

(23) $$p_{B}={\left[\frac{m^{n}}{n^{n}}{\left(p_{A(g)}^{⊖}\right)}^{n}{\left(p_{B(g)}^{⊖}\right)}^{m}\frac{p_{\mathrm{AnBm}(s)}}{p_{\mathrm{AnBm}(s)}^{⊖}}\right]}^{\frac{1}{m+n}}\exp\left(-\frac{nE_{A(g)}+mE_{B(g)}-E_{\mathrm{AnBm}(s)}}{(m+n)RT}\right).$$

Thus, the enthalpy for the above-mentioned chemical reaction is

(24) $$\Delta H_{\mathrm{sub}-\mathrm{rec}}=\frac{nE_{A(g)}+mE_{B(g)}-E_{\mathrm{AnBm}(s)}}{m+n},$$

At this point, a consistent normalization of the enthalpies by converting it to 1 mol leads to

(25) $$\Delta H_{\mathrm{sub}-\mathrm{rec}}^{A}=\frac{nE_{A(g)}+mE_{B(g)}-E_{\mathrm{AnBm}(s)}}{n(m+n)},$$

and

(26) $$\Delta H_{\mathrm{sub}-\mathrm{rec}}^{B}=\frac{nE_{A(g)}+mE_{B(g)}-E_{\mathrm{AnBm}(s)}}{m(m+n)}.$$

For the determination of the prefactor (standard vapor pressure), also the reaction between the gaseous phases has to be considered,

(27) $$A_{n}B_{m}\left(g\right)\to \mathrm{nA}\left(g\right)+\mathrm{mB}\left(g\right)$$

Therefore, the equilibrium pressures of A(g) and B(g) can be calculated based on the above chemical reaction equation. Consequently, the calculation of a complex system with many different gaseous species becomes involved, and we demonstrate it here by looking at examples such as NaCl(s) to the gas phases Na(g) and Cl(g).

### 2.3. Computational Details

This study uses the most stable structure at 0 K, and the basic structures are from the existing Materials Project database [26]. We apply the projector-augmented wave (PAW) method with a 520 eV cutoff energy to represent electron-ion interactions and the generalized gradient approximation (GGA) with the functional proposed by Perdew, Burke, and Ernzerhof (PBE) [27,28] within the plane wave code VASP. Structural and bonding details are listed in Table 1.

For the DFT calculations of the gas phase, we put the single gaseous molecule into a 30×30×30 Å^3^ vacuum box to reduce the interaction between the gaseous molecules to mimic an ideal gas. For the solid structure, we use 2×2×2 or 3×3×3 supercell sizes, the k-point settings depend on the substances and system size, and the Monkhorst–Pack and the Γ centered scheme are used for crystal and gaseous molecules, respectively. Table 2 shows the central results needed for the following steps. In the thermodynamic database FactPS, ${\mathrm{Ni}}_{2}$ and ${\mathrm{Cu}}_{2}$ diatomic gaseous phases exist at the equilibrium state; therefore, their related partial pressures are also explored. Na/Cl(NaCl) and $\mathrm{Zr}/O_{2}\left({\mathrm{ZrO}}_{2}\right)$ express the decomposition reactions of NaCl and ${\mathrm{ZrO}}_{2}$. Na/Cl(NaCl) and $\mathrm{Zr}/O_{2}\left({\mathrm{ZrO}}_{2}\right)$ mean Na(g)/Cl(g) from NaCl(g) and Zr(g) and $O_{2}\left(g\right)$ from ${\mathrm{ZrO}}_{2}\left(g\right)$, respectively.

## 3. Results

### 3.1. Monoatomic Molecule

**Figure 1:**
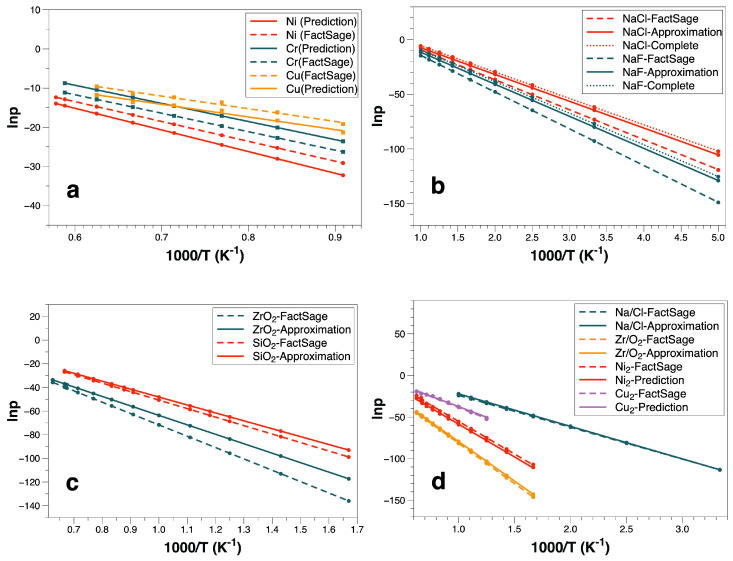
Comparison of the results: (**a**) Monoatomic molecules, (**b**) monoatomic molecule approximation and complete model, (**c**) monoatomic molecule approximation for triatomic molecules, (**d**) chemical reactions.

Figure 1a shows the results of monoatomic molecules: Ni (red solid line), Cr (green solid line), and Cu (orange solid line). The dashed lines represent the results from the calculations with the FactPS database for comparison. In the Arrhenius plot the slope reflects the sublimation enthalpy and the intercept is the prefactor.

The trends (sublimation enthalpy) are nearly the same, and the deviations of sublimation enthalpy are below 10% (Ni: 8.45%, Cr: 1.8%, Cu: 0.1%), and the prefactor accuracy is acceptable (Ni: 6.24%, Cr: 10.45%, Cu: 13.91%). The main reason for the deviation of the prefactor may be the inaccurate calculation of the phonon spectra and the used average frequency.

### 3.2. Monoatomic Molecule Approximation

In this section, we use two different methods to proceed with the theoretical prediction of sublimation functions of NaCl, NaF: (i) Considering the complete description of the molecules and (ii) the monoatomic molecule approximation. Furthermore, we extend the latter to triatomic molecules, ${\mathrm{ZrO}}_{2}$, ${\mathrm{SiO}}_{2}$. The polyatomic molecules’ motion is significantly more complex than for monoatomic molecules, as they have translational, vibrational, and rotational degrees of freedom. To verify the accuracy of the monoatomic molecule approximation, we compare the results for NaCl, NaF with the data considering all degrees of freedom to our approximation in Figure 1b. For a diatomic molecule, the vibrational and rotational degrees of freedom of diatomic molecules are included, i.e., the frequencies of optical branches from phonon spectra, the vibrational frequencies of gaseous molecules and their related rotational motion parameters need to be taken into account. The results of both methods match those obtained from the FactPS database very well. Our approximation influences mainly the prefactor, and the predictions (Approximation: 5.02%, complete: 2.71%) of NaCl are in convincing agreement with the database. Similar results for NaF with similar deviations of the prefactor (approximation: 7.08%, complete: 1.71%) are obtained. Comparisons between our predictions and the database show that the deviations mainly result from the sublimation enthalpy (slope). The bond lengths of NaCl and NaF are calculated using T=0 K DFT calculations, which are found by fitting the energy-volume curves, not considering thermal expansion. Therefore, the accuracy of the bond lengths prediction and the further energy calculations may influence the calculation of the cohesive energy, which in turn affects the further sublimation enthalpy prediction. Nevertheless, our monoatomic molecule approximation leads to reliable predictions.

Furthermore, we have extended the monoatomic molecule approximation to triatomic molecules (${\mathrm{SiO}}_{2}$, ${\mathrm{ZrO}}_{2}$) to demonstrate the precision of the predictions. Figure 1c illustrates our approximation application for the triatomic molecules, and the results are consistent with data obtained from FactSage. The deviations (sublimation enthalpy: 7.06% prefactor: 10.43%) of ${\mathrm{SiO}}_{2}$ are sufficient for many practical applications. However, in contrast to the database, the sublimation enthalpy accuracy of ${\mathrm{ZrO}}_{2}$ is relatively low, and possible reasons are: (i) Due to the instability of these gaseous molecules in reality, the determination of the molecule’s configuration and related structural information is carried out using DFT calculations alone. (ii) The inaccurate energy of the gaseous molecules induces sublimation enthalpy deviations. The following calculations of ${\mathrm{ZrO}}_{2}$ in the next part further support this interpretation.

### 3.3. Chemical Reaction

We study gas phase reactions for ${\mathrm{Ni}}_{2}$, ${\mathrm{Cu}}_{2}$, NaCl and ${\mathrm{ZrO}}_{2}$ as examples, based on the following reactions:

$$2\mathrm{Ni}(s)\to 2\mathrm{Ni}(g)\to {\mathrm{Ni}}_{2}\left(g\right)$$

$$2\mathrm{Cu}(s)\to 2\mathrm{Cu}(g)\to {\mathrm{Cu}}_{2}\left(g\right)$$

NaCl(s)→NaCl(g)→Na(g)+Cl(g)

$${\mathrm{ZrO}}_{2}\left(s\right)\to {\mathrm{ZrO}}_{2}\left(g\right)\to \mathrm{Zr}\left(g\right)+O_{2}\left(g\right)$$

According to Equations (24)–(26), the total enthalpy (sublimation enthalpy + chemical reaction enthalpy) of the above three chemical reactions are obtained,

$$\Delta H_{{\mathrm{Ni}}_{2}}=\frac{E_{{\mathrm{Ni}}_{2}\left(g\right)}-2E_{\mathrm{Ni}(s)}}{1},$$

$$\Delta H_{{\mathrm{Cu}}_{2}}=\frac{E_{{\mathrm{Cu}}_{2}\left(g\right)}-2E_{\mathrm{Cu}(s)}}{1},$$

$$\Delta H_{\mathrm{Na}(g)}=\Delta H_{\mathrm{Cl}(g)}=\frac{E_{\mathrm{Na}(g)}+E_{\mathrm{Cl}(g)}-E_{\mathrm{NaCl}(s)}}{2},$$

$$\Delta H_{\mathrm{Zr}(g)}=\Delta H_{O_{2}\left(g\right)}=\frac{E_{\mathrm{Zr}(g)}+E_{O_{2}\left(g\right)}-E_{{\mathrm{ZrO}}_{2}\left(s\right)}}{2}.$$

The Arrhenius prefactors for these gaseous phases are obtained according to the above chemical reactions. Although other chemical reactions in this process are possible, the unique final equilibrium state between the different gaseous resultants is reached. The source of these gaseous phases is the congruent sublimation process, e.g., the NaCl(s) to NaCl(g) sublimation process, such as Na(g),Cl(g) are all products from NaCl(g). Therefore, two different relevant equilibria play a role, namely between NaCl(s) and NaCl(g) and between NaCl(g) and Na(g),Cl(g). In this paper, subdominant contributions are not considered, such as some elements escaping from the lattice with lower energy and leaving vacancies. Hence, we use $p_{{\mathrm{Ni}}_{2}\left(g\right)}=p_{\mathrm{Ni}(g)}/2$, and $p_{{\mathrm{Cu}}_{2}\left(g\right)}=p_{\mathrm{Cu}(g)}/2$, respectively, for the ${\mathrm{Ni}}_{2}$ and ${\mathrm{Cu}}_{2}$ gas. The dissociation of NaCl and ${\mathrm{ZrO}}_{2}$ is different, where we have by mass conservation $p_{\mathrm{Na}(g)}=p_{\mathrm{Cl}(g)}=p_{\mathrm{NaCl}(g)}$. Figure 1d reveals the precision of our model; especially for ${\mathrm{ZrO}}_{2}$ the deviations of the enthalpy (1.96%) and the prefactor (0.7%) are both below 2%. Furthermore, in this calculation, the energy of ${\mathrm{ZrO}}_{2}\left(g\right)$ is based on Hess’s law reduced, because the two distinct steps are treated as a single step, i.e., step 1: ${\mathrm{ZrO}}_{2}\left(s\right)$ to ${\mathrm{ZrO}}_{2}\left(g\right)$ and step 2: ${\mathrm{ZrO}}_{2}\left(g\right)$ to Zr(g) and $O_{2}\left(g\right)$. We calculate the sublimation enthalpy of Zr(g) and $O_{2}\left(g\right)$ directly using the energy of Zr(g), $O_{2}\left(g\right)$ and ${\mathrm{ZrO}}_{2}\left(s\right)$ without the energy of ${\mathrm{ZrO}}_{2}\left(g\right)$ so that the negative influences from the inaccurate results of energy of ${\mathrm{ZrO}}_{2}\left(g\right)$ disappear. Thus, the inaccuracy of the ${\mathrm{ZrO}}_{2}\left(g\right)$ structural prediction may influence the sublimation enthalpy calculation which leads to a relatively higher deviation. However, the model’s accuracy compared to the database is still convincing.

## 4. Conclusions

In this paper, the possibilities of coupling ab initio calculations, statistical mechanics, and thermodynamic calculations to theoretically investigate the sublimation process, including gas phase reactions, are explored. The following key points can be concluded:

- The comparison of the predictions to thermodynamic databases (FactPS database) has proven that the theoretical prediction of the vapor pressure is promising and precise.
- Theoretical predictions of NaCl and NaF with two different methods reveal the feasibility of the monoatomic molecule approximation. The deviations of the prefactor are 5.02% and 7.08%, respectively. The application to triatomic molecules (${\mathrm{ZrO}}_{2}$ and ${\mathrm{SiO}}_{2}$) indicates the benefit for complex situations, especially ${\mathrm{SiO}}_{2}$, where the deviations of the sublimation enthalpy and the prefactor are 7.06% and 10.43%, respectively.
- The additional exploration of the formation of ${\mathrm{Ni}}_{2}\left(g\right)$, ${\mathrm{Cu}}_{2}\left(g\right)$, Na(g), Cl(g), Zr(g) and $O_{2}\left(g\right)$ indicates the rationality of our theoretical calculation to explain further chemical reactions. Except for the predicted prefactor of Na/Cl(g) and ${\mathrm{Ni}}_{2}$, the deviations are below 5%.
- The determination of unknown gaseous molecule structures, the approximation of the molecules’ motion, and inaccuracies of the thermodynamic database may lead to deviations.

## Figures and Tables

**Table 1:** Structural information used for the DFT calculations.

Substance	Crystal System	Space Group	Number	Chemical Bond
Ni	cubic	Fm3m	225	Metallic
Cr	cubic	Im3m	229	Metallic
Cu	cubic	Fm3m	225	Metallic
NaCl	cubic	Fm3m	225	Ionic
NaF	cubic	Fm3m	225	Ionic
${\mathrm{ZrO}}_{2}$	monoclinic	P21/c	14	Ionic
${\mathrm{SiO}}_{2}$	tetragonal	I42d	122	Covalent

**Table 2:** Comparison of the prediction to the thermodynamic database results.

Substance	FactSage		Monoatomic Molecule Approximation		Complete Model	
	$H_{\mathrm{sub}}\left(J\right)$	A	$H_{\mathrm{sub}}\left(J\right)$	A	$H_{\mathrm{sub}}\left(J\right)$	A
Ni	−50.677	16.94	−	−	−55.354	18.068
${\mathrm{Ni}}_{2}$	−76.544	20.81	−	−	−77.080	18.02
Cr	−47.163	16.59	−	−	−46.420	18.55
Cu	−39.847	14.88	−	−	−39.795	16.95
${\mathrm{Cu}}_{2}$	−56.628	18.41	−	−	−54.532	18.02
NaCl	−27.404	18.069	−24.520	17.162	−24.117	18.559
Na/Cl(NaCl)	−38.358	14.464	−39.139	16.38	−	−
NaF	−33.624	19.271	−29.364	17.906	−29.000	19.6
${\mathrm{ZrO}}_{2}$	−93.144	21.84	−80.045	17.61	−	−
Zr/$O_{2}\left({\mathrm{ZrO}}_{2}\right)$	−97.014	15.56	−95.113	15.67	−	−
${\mathrm{SiO}}_{2}$	−71.753	20.99	−66.9	18.8	−	−

## Data Availability

Not applicable.

## Abbreviations

The following abbreviations are used in this manuscript:

SOFC	Solid oxide fuel cell
KEMS	Knudsen effusion mass spectrometry
DFT	Density functional theory
VASP	Vienna ab initio simulation package
PAW	Project augmented wave
PBE	Perdew, Burke and Ernzerhof
GGA	Generalized gradient approximation
